# Early Postoperative Endothelial Cell Density Predicts Graft Survival After Keratoplasty: A Systematic Review and Meta-Analysis

**DOI:** 10.7759/cureus.100799

**Published:** 2026-01-05

**Authors:** Haidar Bonajmah, Faisal Aljassar

**Affiliations:** 1 Ophthalmology, Kuwait Institute for Medical Specialization, Kuwait City, KWT; 2 Department of Ophthalmology, Mohamed Abdulrahman Al Bahar Eye Centre, Ibn Sina Hospital, Kuwait City, KWT

**Keywords:** corneal graft, corneal transplantation, endothelial cell density, keratoplasty, meta-analysis, systematic review

## Abstract

Long-term graft clarity after corneal transplantation depends on sustained endothelial survival, yet the prognostic role of early postoperative endothelial metrics has not been clearly synthesized. We therefore reviewed longitudinal clinical evidence and quantitatively summarized the relationship between early postoperative endothelial status and later endothelial failure-related graft failure across common keratoplasty techniques. Overall, lower endothelial reserve measured in the early postoperative period consistently aligned with higher subsequent failure risk, with suggestive signals that risk may rise steeply below certain low-density ranges, while a minority of grafts can remain clear despite very low counts. Limited data also indicate that some endothelial morphological features may add prognostic value beyond cell density. These findings support using early postoperative endothelial assessment as part of practical risk stratification and follow-up planning, alongside measures aimed at preserving endothelial reserve to help extend graft longevity.

## Introduction and background

Corneal endothelial dysfunction is a major cause of corneal blindness and a leading indication for keratoplasty [1]. Over the past two decades, penetrating keratoplasty (PK) has been largely replaced by posterior lamellar techniques: Descemet stripping automated endothelial keratoplasty (DSAEK) and Descemet membrane endothelial keratoplasty (DMEK), which provide faster visual recovery, less rejection, and better refractive stability than full-thickness grafting [2]. Long-term graft clarity depends on donor endothelial survival: because adult corneal endothelial cells have limited proliferative capacity in vivo under physiological conditions, progressive endothelial cell density (ECD) loss remains a key driver of late endothelial failure after PK and endothelial keratoplasty. Emerging regenerative strategies (e.g., ROCK inhibition) may enhance endothelial cell migration and proliferative responses in selected settings, but do not yet negate the clinical importance of endothelial reserve for long-term graft survival [3].

Donor selection currently emphasises preoperative ECD, typically requiring ≥2200-2400 cells/mm² [4], but early postoperative ECD also reflects intraoperative trauma, postoperative events (e.g., dislocation, rebubbling), and the host environment [5]. Thus, early ECD may better capture the “effective” endothelial reserve, yet there is no consensus on the most informative timepoint, threshold, or effect size, and postoperative ECD is rarely used formally in prognostication. In routine practice, ECD is most commonly quantified from central corneal specular microscopy (or confocal microscopy in some settings), which provides a cells/mm² estimate based on automated or manual cell counting and is widely used to monitor endothelial health before and after keratoplasty.

Individual cohorts suggest that lower six-month ECD after PK or endothelial keratoplasty is associated with a higher risk of endothelial failure [6-8], while other series mainly describe ECD trajectories and determinants such as iris damage, sutured intraocular lenses, surgical difficulty, or rebubbling [9-11]. However, the consistency of the association between early ECD and failure is still unknown.

We therefore conducted a systematic review and meta-analysis to assess the prognostic value of early postoperative ECD for endothelial failure-related graft failure. Our objectives were to quantify the association between six-month ECD and subsequent endothelial failure using hazard ratios standardised per 100 cells/mm² lower ECD, summarise evidence on categorical ECD thresholds, very low ECD levels, and early percentage ECD loss; explore the additional prognostic contribution of endothelial morphology; and describe clinical and surgical determinants of low postoperative ECD across PK, DSAEK, and DMEK.

## Review

Methods

Protocol and Registration

The review was conducted in accordance with the Preferred Reporting Items for Systematic Reviews and Meta-Analyses (PRISMA) statement and methodological guidance from the Cochrane Handbook for Systematic Reviews [12,13]. No substantive deviations from the prespecified eligibility criteria, outcomes, or primary synthesis approach were made.

Information Sources and Search Strategy

We searched PubMed (MEDLINE), Web of Science, Scopus, and CENTRAL from inception to May 2025 using controlled vocabulary and free-text terms for keratoplasty procedures, endothelial metrics, and graft failure; full database-specific strategies are provided in the Appendix.

Eligibility Criteria

We included longitudinal human studies (prospective or retrospective cohorts, and randomized trials when analysed as cohorts) that enrolled patients undergoing corneal transplantation (PK, DSAEK, DMEK, or other endothelial keratoplasty) for any indication, measured postoperative central endothelial cell density (ECD) at one or more early timepoints (preferably one to three months and/or six months), evaluated the association between postoperative ECD (and, secondarily, postoperative hexagonality (HEX) or coefficient of variation (CV)) and subsequent endothelial failure-related graft failure or regraft, and reported effect estimates (hazard ratios, odds ratios, or risk ratios) or data permitting their interpretation.

We excluded studies that were non-human, cadaveric, or in vitro; cross-sectional designs without longitudinal follow-up; case reports, case series with <10 eyes, narrative or systematic reviews, editorials, letters, conference abstracts without full data, clinical guidelines, and book chapters. We also excluded studies that lacked postoperative ECD measurements, did not distinguish endothelial failure from other causes of graft failure, or were not available in English.

Selection Process

All records retrieved from the databases were imported into Rayyan, and duplicates were removed manually. Two reviewers independently screened titles and abstracts for potential relevance. Articles deemed potentially eligible by either reviewer proceeded to full-text assessment. The same reviewers then evaluated full texts against the predefined eligibility criteria to determine inclusion in the qualitative synthesis and meta-analysis. Disagreements at any stage were resolved by discussion; if consensus could not be reached, a third reviewer acted as arbiter.

Data Collection Process, Data Items, and Outcomes

Data extraction was performed independently by two reviewers using a piloted Excel sheet tailored to prognostic studies of corneal transplantation. For each study, we recorded: first author, year, country, study design, procedure type (PK, DSAEK, DMEK), sample size (eyes), and length of follow-up. We extracted the exact definition of endothelial failure-related graft failure or secondary endothelial failure.

Index-test data comprised postoperative ECD (cells/mm²) at all reported timepoints, particularly one to three months and six months, together with measures of endothelial morphology (HEX, CV) when available. For eligible prognostic analysis, we extracted the effect measure (hazard ratio, odds ratio, or risk ratio) for postoperative ECD, along with their 95% confidence intervals and the variables included in multivariable models. When ECD effects were expressed per 500-cells/mm² decrement or other scales, we recorded the original scale for later standardisation. Any discrepancies in data abstraction were resolved by consensus, with involvement of a third reviewer when necessary.

Risk-of-Bias Assessment

Two investigators independently assessed the risk of bias of each included study using the Newcastle-Ottawa Scale (NOS) for cohort studies, evaluating three domains: Selection (0-4 stars), Comparability (0-2 stars), and Outcome (0-3 stars) [14]. Total scores (0-9 stars) were used to classify overall risk of bias as low (8-9), moderate (6-7), or high (≤5). Differences in scoring were discussed until agreement was reached.

Statistical Analysis

All statistical analyses were performed using R version 4.3.3 with the metafor package [15]. For each study, hazard ratios and corresponding 95% confidence intervals were log-transformed, and the standard errors were derived from the reported confidence limits. When effect estimates were presented per 500 cells/mm² decrease in ECD, we rescaled them to a common metric of per 100 cells/mm² lower postoperative ECD by raising the HR and its confidence limits to the power of 0.2. The primary meta-analysis pooled adjusted hazard ratios for the six-month postoperative ECD and endothelial failure-related graft failure using an inverse-variance common-effect model. Statistical heterogeneity was quantified using the between-study variance (τ²) and the I² statistic. Secondary analyses synthesised categorical ECD thresholds and HEX/CV effects narratively, given the small number of studies and heterogeneous definitions. An omnibus p-value <0.05 was considered statistically significant. Assessment of small-study effects/publication bias using funnel plots or formal tests (e.g., Egger’s) was not considered informative given the small number of studies (n = 4) contributing to the primary meta-analysis. Given the small number of studies, we prespecified a sensitivity analysis using a random-effects model to verify the reliability of the pooled estimate to between-study variance assumptions.

Results

Study Selection

**Figure 1:**
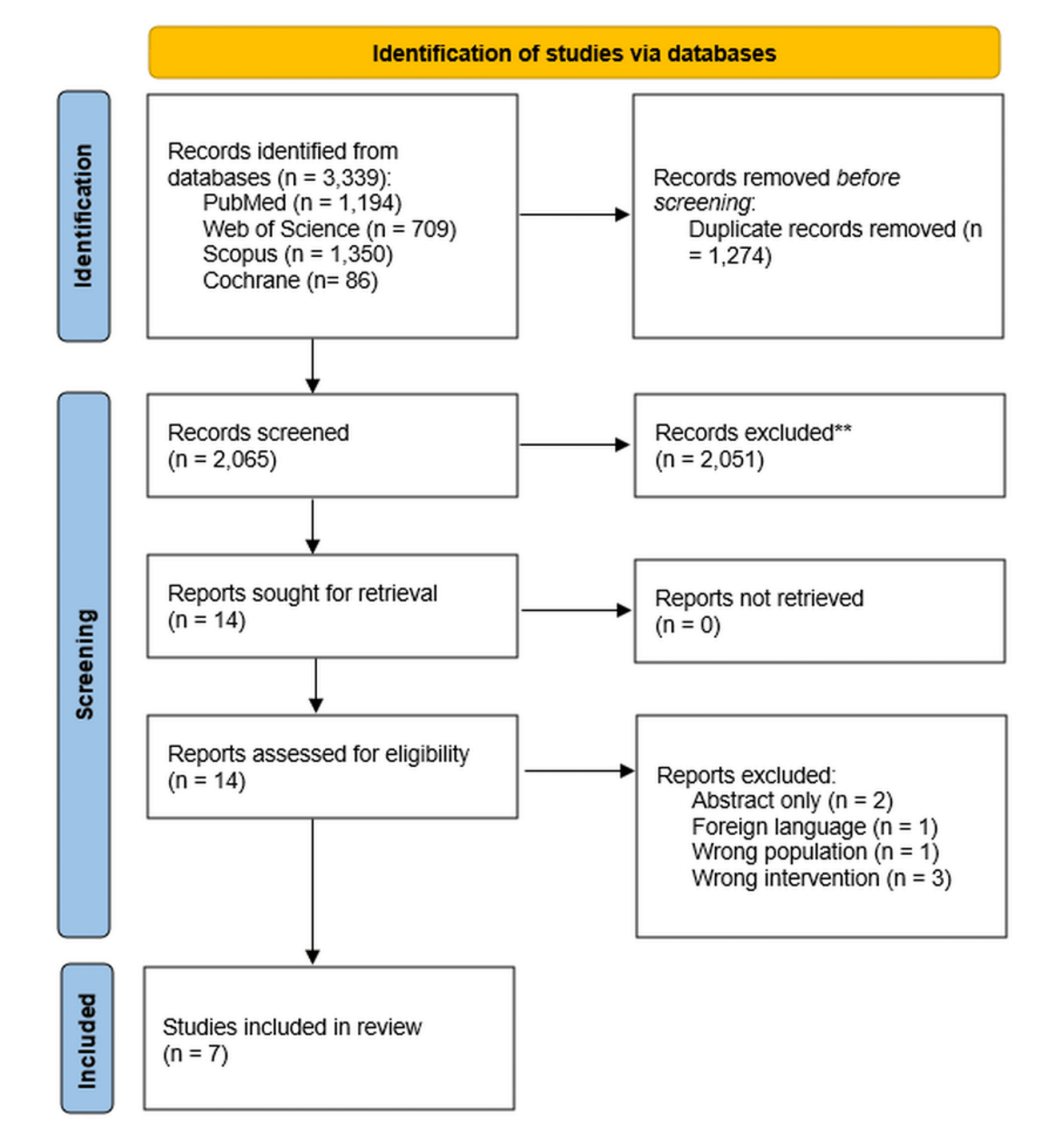
PRISMA flowchart

Our database search identified 3,339 records in total (PubMed = 1,194; Web of Science = 709; Scopus = 1,350; Cochrane = 86). After removing 1,274 duplicates, 2,065 unique records remained for title-and-abstract screening. Of these, 2,051 were considered irrelevant and excluded, leaving 14 articles for full-text assessment. Detailed eligibility evaluation excluded seven reports (two abstract-only publications, one foreign-language article, one with the wrong population, and three with the wrong intervention). Ultimately, seven eligible studies fulfilled all criteria and were included in the review [6-11,16]. The study selection process is detailed in the PRISMA flow diagram (Figure 1).

Study Characteristics

Seven longitudinal cohort studies met the inclusion criteria: two PK cohorts (Lass et al., 2010 [16]; Benetz et al., 2013 [8]), one multicentre DSAEK trial (Patel et al., 2019 [7]), and four DSAEK/DMEK single-centre series (Ishii et al., 2016 [10]; Vasiliauskaitė et al., 2021 [6]; Chaussard et al., 2022 [11]; Viola et al., 2024 [9]). Across studies, follow-up ranged from 12 months to more than 10 years. All studies reported postoperative ECD; four reported time-to-event models linking early postoperative ECD to endothelial failure-related graft failure (Lass et al., 2010 [16]; Benetz et al., 2013 [8]; Patel et al., 2019 [7]; Vasiliauskaitė et al., 2021 [6]), and one additionally modelled postoperative HEX and CV as predictors (Benetz et al., 2013 [8]). The remaining three studies focused on ECD trajectories and determinants (Ishii et al., 2016 [10]; Chaussard et al., 2022 [11]; Viola et al., 2024 [9]) and on clinical outcomes such as graft survival, visual acuity, and rebubbling. Detailed summary and characteristics of included studies are demonstrated in Table 1.

**Table 1:** Summary and baseline characteristics of the included studies PK: penetrating keratoplasty; DSAEK: Descemet stripping automated endothelial keratoplasty; DMEK: Descemet membrane endothelial keratoplasty; ECD: endothelial cell density; ECC: endothelial cell count; HEX: percentage of hexagonal endothelial cells; CV: coefficient of variation in endothelial cell area; TS-IOL: transscleral-sutured intraocular lens; LEGF: late endothelial graft failure; CCT: central corneal thickness; HR: hazard ratio; ECL: endothelial cell loss

Study (year)	Design	Procedure and main indication(s)	Sample size (eyes)	Follow-up	Index test(s)	Outcome(s)	Effect measures reported for prognosis
Vasiliauskaitė et al., 2021 [6]	Retrospective cohort	DMEK	585 eyes	Five years	Six-month central ECD (quartiles and continuous), plus preoperative ECD	Endothelial graft failure / secondary failure (persistent edema or regraft after initial clarity); long-term ECD	Cox models reporting HR per 500 cells/mm² lower six-month ECD (converted to HR per 100 cells/mm²); survival by ECD quartiles and low-ECD subgroups
Patel et al., 2019 [7]	Prospective multicentre cohort	DSAEK	1,223 eyes (1,209 non-failures, 14 late endothelial graft failures)	Up to five years	Central donor ECD and six- and 12-month postoperative ECD	Late endothelial graft failure (LEGF) – irreversible endothelial failure after initial clarity	Cox models with HR per 500 cells/mm² lower six-month ECD (converted in meta-analysis to HR per 100 cells/mm²); additional models adjusted for surgical complications
Benetz et al., 2013 [8]	Prospective cohort	PK	72 eyes with baseline and follow-up morphometry	Up to five years	Six-month ECD, plus six-month HEX (%) and CV from central specular images	Endothelial graft failure, as in Lass et al.	Multivariable Cox models: HR per 100 cells/mm² lower six-month ECD, HR per 10% lower HEX, HR per 0.10 higher CV
Viola et al., 2024 [9]	Retrospective cohort	DMEK	65 eyes	36 months	Donor ECC and serial postoperative ECC (one to 36 months), modelled with a biexponential decay; comparison of DMEK alone vs. DMEK + cataract	Long-term predicted endothelial cell loss, rebubbling, visual acuity, and CCT	No time-to-failure HRs; provides parameters of ECC decay curves and group comparisons (ECL over time, predicted time to ECC thresholds)
Ishii et al., 2016 [10]	Retrospective cohort	DSAEK	225 eyes in 198 patients	Up to 36 months	Donor ECD and serial postoperative graft ECD (three, six, 12, 24, 36 months)	Graft failure due to endothelial decompensation; longitudinal ECD decline	Cox regression for graft failure (main predictors: iris damage, prior surgery, TS-IOL, etc.); ECD is mainly treated as an outcome, not the primary prognostic index test
Chaussard et al., 2022 [11]	Retrospective cohort	DMEK	103 eyes (95 patients)	12 months	Donor ECD and six- and 12-month central ECD, analysed overall and dichotomised at 1000 cells/mm²; HEX/CV not modelled	Low postoperative ECD (<1000 vs ≥1000 cells/mm²), graft failure, and repeat keratoplasty, rebubbling	Linear and logistic regression for predictors of the six- and 12-month ECD and of ECD <1000 cells/mm²; no continuous ECD→failure HR, but failure rates reported by ECD category and surgical difficulty
Lass et al., 2010 [16]	Prospective cohort	PK	500 grafts with central ECD data (17 endothelial failures, 483 non-failures)	Up to five years	Central donor ECD and six-month postoperative central ECD (specular microscopy)	Endothelial graft failure (loss of central clarity ≥3 months due to endothelial decompensation; regraft or persistent edema)	Cox models giving HR per 100 cells/mm² lower six-month ECD for endothelial failure

Risk-of-Bias Assessment

Most studies were judged to be at low or only moderate risk of bias. Lass et al. (2010), Patel et al. (2019), and Vasiliauskaitė et al. (2021) scored 9/9 stars with low risk of bias, reflecting representative cohorts, clear exposure and outcome definitions, appropriate multivariable adjustment, and long, well-completed follow-up [6,7,16]. Benetz et al. (2013) scored slightly lower (8/9, low-moderate risk) because it analysed only a morphometry subset of the parent cohort, introducing some selection concern [8]. Ishii et al. (2016), Chaussard et al. (2022), and Viola et al. (2024) (all 7/9, moderate risk) were downgraded mainly due to retrospective single-centre designs, smaller sample sizes, and shorter or less comprehensive follow-up and outcome capture, rather than major problems with exposure or outcome measurement [9-11] (Table 2).

**Table 2:** Risk-of-bias assessment for the included studies

Study	Design	Selection (0–4★)	Comparability (0–2★)	Outcome (0–3★)	Total (0–9★)	Overall risk of bias
Vasiliauskaitė et al., 2021 [6]	Retrospective cohort	★★★★	★★	★★★	9	Low
Patel et al., 2019 [7]	Prospective multicentre cohort	★★★★	★★	★★★	9	Low
Benetz et al., 2013 [8]	Prospective cohort	★★★	★★	★★★	8	Low–moderate
Viola et al., 2024 [9]	Retrospective cohort	★★★	★★	★★	7	Moderate
Ishii et al., 2016 [10]	Retrospective cohort	★★★	★★	★★	7	Moderate
Chaussard et al., 2022 [11]	Retrospective cohort	★★★	★★	★★	7	Moderate
Lass et al., 2010 [16]	Prospective cohort	★★★★	★★	★★★	9	Low

Primary Outcome: Six-Month Postoperative ECD and Endothelial Failure-Related Graft Failure

**Figure 2:**
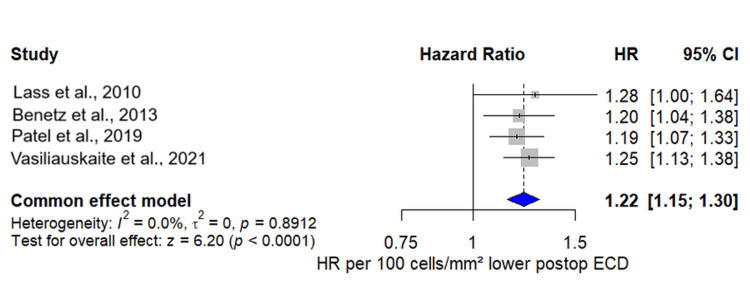
Forest plot showing study-specific and pooled hazard ratios for endothelial failure-related graft failure per 100 cells/mm² lower six-month postoperative endothelial cell density Pooled studies are Vasiliauskaite et al. [6], Patel et al. [7], Benetz et al. [8], and Lass et al. (2010) [16].

Four studies provided hazard ratios relating the six-month postoperative ECD to subsequent endothelial failure-related graft failure (Figure 2). After standardising all estimates to a common scale of HR per 100 cells/mm² lower six-month ECD, each study showed a positive association between lower ECD and higher failure risk. Individual HRs ranged from 1.19 to 1.28 per 100-cell decrement. When pooled in a common-effect model, every 100 cells/mm² reduction in the six-month ECD was associated with a 22% increase in the risk of endothelial failure (pooled HR 1.22, 95% CI 1.15-1.30), with no evidence of between-study heterogeneity (I² = 0%). This effect was consistent across PK, DSAEK, and DMEK procedures and across multicentre and single-centre designs. In sensitivity analysis using a random-effects model, the pooled association was unchanged to materially similar, and heterogeneity remained negligible (τ² = 0; I² = 0%), confirming the reliability of our findings.

Although several studies reported ECD at earlier visits (one to three months), none provided time-to-event effect estimates using ECD at those timepoints as a continuous prognostic predictor. Descriptively, early postoperative ECD values were already reduced by approximately 30-40% from donor levels and continued to decline between three and six months, but the prognostic analyses consistently used the six-month measurement as the clinically stable index [6-8,16].

Three studies further explored threshold-based risks. In the DSAEK trial of Patel et al. (2019), the cumulative incidence of late endothelial graft failure by five years was 6.5% among eyes with six-month ECD <1200 cells/mm², compared with ≤0.6% in those with ECD ≥1200 cells/mm², suggesting a sharp risk inflection around this level [7]. In the DMEK series of Vasiliauskaitė et al. (2021), most endothelial failures occurred in the lowest quartile of the six-month ECD, and within that group, eyes with ECD ≤828 cells/mm² had substantially lower five-year survival (0.79) than those with higher ECD, which had survival probabilities near 1.0 [6]. Chaussard et al. (2022), using a <1000 vs. ≥1000 cells/mm² dichotomy at six and 12 months, found that low ECD was strongly associated with earlier graft failure and with a history of technically difficult surgery [11].

Early percentage ECD loss from baseline to six months was also evaluated in several studies [6-8,16]. Unadjusted analyses suggested that greater early loss was associated with higher failure risk; however, in multivariable models that included both six-month ECD and early loss, the absolute six-month ECD level remained the dominant predictor, and early loss contributed little additional information.

Several cohorts reported how often very low ECD levels were reached without frank failure. In the DMEK study by Vasiliauskaitė et al. (2021), a small proportion of grafts with ECD <500 cells/mm² at five years remained clear, illustrating that although very low ECD markedly increases risk, graft clarity can occasionally be maintained even at these levels [6].

Secondary Findings: Endothelial Morphology (HEX, CV)

Only Benetz et al. (2013) provided time-to-event analyses for postoperative endothelial morphology [8]. After adjustment for the six-month ECD, a lower percentage of hexagonal cells at six months remained independently associated with graft failure: each 10% reduction in HEX increased the hazard of endothelial failure more than twofold. By contrast, the coefficient of variation in cell area at six months showed no clear association, with wide confidence intervals.

Secondary Findings: Determinants of Low Postoperative ECD

Several studies provided insight into factors that drive postoperative endothelial loss and thereby indirectly influence failure risk. In the DSAEK cohort of Ishii et al. (2016), higher pre-existing iris damage scores, transscleral sutured IOLs, multiple previous intraocular surgeries, surgeon inexperience, and postoperative rebubbling were all associated with more rapid ECD decline and worse graft survival; pre-existing iris damage remained a strong predictor even in “uncomplicated” cases [10]. Chaussard et al. (2022) identified “difficult surgery” (especially challenging graft dissection or unfolding) as the main independent determinant of low six- and 12-month ECD and of ECD <1000 cells/mm² [11]. Patel et al. (2019) showed that operative complications, graft dislocation, and donor diabetes were all associated with lower six-month ECD, and that operative complications remained an independent predictor of late endothelial failure after adjustment for ECD [7]. Viola et al. (2024), studying preloaded DMEK, found no short-term difference in ECD loss between stand-alone DMEK and triple procedures, but mathematical modelling suggested somewhat steeper late-phase ECD decline in eyes combined with cataract surgery [9].

Other Outcomes: Visual Acuity, Graft Survival, and Postoperative Events

Functional outcomes were consistently favourable in the modern endothelial keratoplasty series. Ishii et al. (2016) [10], Chaussard et al. (2022) [11], and Viola et al. (2024) [9] all reported substantial improvements in best-corrected visual acuity by six to 12 months, with mean postoperative acuities approaching 0.3 logMAR or better, despite ongoing ECD loss. None of the studies demonstrated a clear quantitative link between postoperative ECD and medium-term visual acuity among surviving grafts, suggesting that ECD in the ranges typically observed after contemporary EK primarily affects the risk of late failure rather than early visual function.

Overall graft survival was high across procedures, but secondary endothelial failure accounted for most late graft losses [6-8,11,16]. In Vasiliauskaitė et al. (2021), endothelial failures were rare outside the lowest six-month ECD strata [6]. In Chaussard et al. (2022), approximately one in ten grafts failed by 12 months, again concentrated among eyes with low ECD and difficult surgery [11]. Rebubbling or graft detachment occurred in roughly one-third of DMEK eyes in several series [6,9-11]; these events were consistently associated with lower subsequent ECD, although not always independently with failure once ECD was accounted for [7,10]. Other complications, such as rejection, cystoid macular edema, or transient intraocular pressure elevations, were infrequent and seldom led directly to irreversible endothelial failure [7,8,10,11,16].

Discussion

Principal Findings

Our findings show that early postoperative endothelial cell density is a powerful and consistent prognostic marker across modern corneal transplantation techniques. Pooling four adjusted Cox models, every 100 cells/mm² lower six-month ECD increased the hazard of endothelial failure-related graft failure by about 22%, with virtually no between-study heterogeneity. This association was stable across PK, DSAEK, and DMEK and across single- and multicentre cohorts, suggesting that six-month ECD functions as a generic indicator of “effective” endothelial reserve rather than a procedure-specific signal. The effect size is comparable to the prognostic gradient reported for donor ECD in long-term PK cohorts, where a 500 cells/mm² lower donor ECD increased late endothelial failure risk by about 1.6-fold [17].

Clinical Interpretation and Thresholds

These results emphasise that long-term outcomes reflect not only donor starting reserve but also survival through the early postoperative “bottleneck” of surgical manipulation, detachment/rebubbling, and host-related stressors. Consistent with longitudinal EK literature, ECD declines steeply early (often ~40-50% within the first postoperative year) before entering a slower long-term attrition phase [18,19]. The 6-month timepoint plausibly represents a clinically useful quasi-steady state, after early events have occurred and clarity is restored, yet before longer-term attrition dominates, making it a practical point for risk stratification. Mechanistically, lower ECD implies reduced pump reserve and a smaller physiologic buffer against subsequent insults such as rejection, transient oedema, or intraocular pressure spikes [20].

Threshold findings across included cohorts support a steepening risk gradient at lower cell counts rather than a single universal cut-off. In CPTS, a six-month DSAEK ECD <1200 cells/mm² was associated with an order-of-magnitude higher five-year failure risk than higher counts [7], while Vasiliauskaitė et al. reported reduced DMEK survival in the lowest six-month ECD quartile (≤828 cells/mm²) [6]. Long-term DSEK and PK cohorts similarly describe many surviving grafts near 700-900 cells/mm² and occasional clear grafts even below 500 cells/mm² [16,21,22]. Taken together, postoperative ECD is best interpreted as a continuous risk factor, while values below ~800-1000 cells/mm² at six months reasonably flag a higher-risk trajectory.

Contemporary Context and Future Directions

Emerging regenerative approaches further support biological plausibility. ROCK inhibition (e.g., ripasudil or Y-27632) can enhance endothelial cell migration, proliferative responses, and pump-function recovery in experimental and early clinical settings, and has been associated with reduced endothelial loss or improved endothelial metrics in selected scenarios such as FECD/Descemet-stripping-only pathways [23,24]. These developments do not diminish the prognostic role of early endothelial reserve; rather, they highlight modifiable pathways that could be targeted to preserve or restore reserve and potentially reduce late endothelial failure risk [7].

Recent contemporary long-term EK datasets, including 10-year follow-up cohorts and registry analyses, provide complementary context by demonstrating sustained endothelial attrition over time and reinforcing endothelial reserve as a long-horizon constraint on graft longevity, even when six-month ECD is not modelled as a formal hazard predictor [25-27].

Future research should prioritise multivariable prognostic models integrating six-month ECD with clinical predictors such as glaucoma, prior ocular surgery, rejection history, and surgical difficulty. Such models should be developed and externally validated across PK, DSAEK, and DMEK in large registries and translated into practical tools that estimate absolute five-to-10-year failure probabilities. In parallel, trials of endothelial-preserving strategies (including ROCK-inhibitor adjuncts, modified insertion devices, and gentler unfolding techniques) should routinely include postoperative ECD and long-term endothelial failure as prespecified endpoints. More granular assessment of morphology (HEX, CV) and function, potentially supported by automated or AI-assisted analysis, may clarify whether structural abnormalities add prognostic information beyond density alone.

Limitations

This review has several limitations. First, only four studies provided time-to-event estimates for continuous postoperative ECD, limiting precision for procedure- or indication-specific subgroup analyses. Second, although pooled hazard ratios were adjusted, covariate sets differed across studies, and residual confounding (notably by surgical complexity, rejection episodes, and glaucoma) remains possible. Third, definitions of endothelial failure and the exact timing of ECD measurements were broadly similar but not identical, and measurement error in specular microscopy could attenuate true associations. Fourth, most data arose from FECD or moderate-risk pseudophakic/aphakic oedema, so generalisability to high-risk grafts, paediatric patients, or severe ocular surface disease is uncertain. Finally, publication and language bias cannot be excluded because non-English and unpublished studies were not included.

Clinical Implications

Clinically, these findings support incorporating six-month ECD into routine postoperative assessment and patient counselling. Eyes with higher ECD at this time point appear to have relatively low near-term failure risk, whereas markedly reduced counts warrant closer monitoring and proactive management of modifiable risks (e.g., intraocular pressure spikes and inflammation), with early consideration of restorative or surgical rescue strategies when clinically indicated. Because some grafts remain clear even at very low ECD, decisions about re-intervention should not rely on ECD alone but be anchored to symptoms, visual acuity, pachymetry, and clinical course.

## Conclusions

This systematic review highlights the importance of early postoperative endothelial evaluation as a meaningful component of long-term graft care following corneal transplantation. Postoperative endothelial measures reflect the combined effects of donor factors, surgical technique, and postoperative stress on the graft and therefore provide a clinically relevant window into future graft health. Incorporating these early assessments into routine practice may support more individualized surveillance strategies, timely intervention when endothelial reserve appears compromised, and benchmarking of surgical quality across procedures. As endothelial-preserving techniques and therapies continue to evolve, aligning postoperative monitoring with these advances offers a practical pathway toward improving graft durability and reducing the long-term burden of endothelial failure and repeat transplantation.

## Appendices

**Table 3:** Search strategy

Database	Search query	Results
PubMed	("Corneal Transplantation" OR "Keratoplasty, Penetrating" OR "Descemet Stripping Endothelial Keratoplasty" OR "Descemet Membrane Endothelial Keratoplasty" OR keratoplast* OR "corneal transplant*" OR "corneal graft*" OR DSAEK OR DMEK OR DLEK OR "endothelial keratoplast*" OR "Descemet stripping" OR "Descemet membrane") AND ("Endothelium, Corneal" OR "endothelial cell densit*" OR "endothelial cell count*" OR "endothelial cell loss" OR ECD) AND ("graft failure*" OR "graft survival" OR "graft loss" OR regraft* OR "late endothelial failure" OR "endothelial failure" OR "endothelial decompensation" OR "secondary graft failure" OR "secondary failure")	1194
Scopus	TITLE-ABS-KEY (("Corneal Transplantation" OR "Keratoplasty, Penetrating" OR "Descemet Stripping Endothelial Keratoplasty" OR "Descemet Membrane Endothelial Keratoplasty" OR keratoplast* OR "corneal transplant*" OR "corneal graft*" OR DSAEK OR DMEK OR DLEK OR "endothelial keratoplast*" OR "Descemet stripping" OR "Descemet membrane") AND ("Endothelium, Corneal" OR "endothelial cell densit*" OR "endothelial cell count*" OR "endothelial cell loss" OR ECD) AND ("graft failure*" OR "graft survival" OR "graft loss" OR regraft* OR "late endothelial failure" OR "endothelial failure" OR "endothelial decompensation" OR "secondary graft failure" OR "secondary failure"))	1350
Web of science	(("Corneal Transplantation" OR "Keratoplasty, Penetrating" OR "Descemet Stripping Endothelial Keratoplasty" OR "Descemet Membrane Endothelial Keratoplasty" OR keratoplast* OR "corneal transplant*" OR "corneal graft*" OR DSAEK OR DMEK OR DLEK OR "endothelial keratoplast*" OR "Descemet stripping" OR "Descemet membrane") AND ("Endothelium, Corneal" OR "endothelial cell densit*" OR "endothelial cell count*" OR "endothelial cell loss" OR ECD) AND ("graft failure*" OR "graft survival" OR "graft loss" OR regraft* OR "late endothelial failure" OR "endothelial failure" OR "endothelial decompensation" OR "secondary graft failure" OR "secondary failure")) (Topic)	709
Cochrane	("Corneal Transplantation" OR "Keratoplasty, Penetrating" OR "Descemet Stripping Endothelial Keratoplasty" OR "Descemet Membrane Endothelial Keratoplasty" OR keratoplast* OR "corneal transplant*" OR "corneal graft*" OR DSAEK OR DMEK OR DLEK OR "endothelial keratoplast*" OR "Descemet stripping" OR "Descemet membrane") AND ("Endothelium, Corneal" OR "endothelial cell densit*" OR "endothelial cell count*" OR "endothelial cell loss" OR ECD) AND ("graft failure*" OR "graft survival" OR "graft loss" OR regraft* OR "late endothelial failure" OR "endothelial failure" OR "endothelial decompensation" OR "secondary graft failure" OR "secondary failure")	86
